# The Interpersonal Antecedents of Attachment Security in Early Adulthood

**DOI:** 10.3390/children12020255

**Published:** 2025-02-19

**Authors:** Julie A. Blake, James G. Scott, Jake M. Najman, Hannah J. Thomas

**Affiliations:** 1 Child Health Research Centre, The University of Queensland, South Brisbane, QLD 4101, Australia; james.scott@health.qld.gov.au; 2 Child and Youth Mental Health Service, Children’s Health Queensland, South Brisbane, QLD 4101, Australia; 3 Queensland Centre for Mental Health Research, Wacol, QLD 4076, Australia; hannah.thomas@uq.edu.au; 4 School of Public Health, Faculty of Medicine, The University of Queensland, Herston, QLD 4006, Australia; j.najman@uq.edu.au

**Keywords:** relationships, parenting, social support, family, friendship, childhood

## Abstract

**Background/Objectives**: The relevance of adult attachment security for physical and emotional wellbeing is increasingly evident. Developing a better understanding of the interpersonal antecedents of secure attachment in childhood and adolescence could enable opportunities for its promotion when attachment styles are more easily modifiable. **Methods**: Data from 3648 participants in a longitudinal birth cohort study were examined. At 21 years, participants completed the confidence (in self and others) subscale of the Attachment Style Questionnaire, a measure of attachment security. Path analysis was used to longitudinally examine the influence of maternal and child-reported interpersonal variables at birth, 5, and 14 years on attachment security in early adulthood. **Results**: Two pathways were identified. Firstly, child and family social relations at birth and 5 years predicted attachment security via the number of the child’s close friends at 14 years (β = 0.11, *p* < 0.001). Secondly, attuned caregiving at 14 years predicted attachment security via the recalled experiences of parental care up to 16 years, measured at 21 years (β = 0.28, *p* < 0.001). Greater adolescent family satisfaction directly predicted increased attachment security in early adulthood (β = 0.10, *p* < 0.001). **Conclusions**: Child and family social relationships from birth and throughout childhood and adolescence, along with attuned caregiving, led to increased attachment security in early adulthood. Public health and policy initiatives aimed at strengthening social support systems for caregivers and their children throughout childhood, and increasing the uptake of parenting programmes aimed at strengthening attuned caregiving, may lead to long-term improvements in the attachment security of offspring.

## 1. Introduction

Secure attachment is an important part of healthy social–emotional development and self-regulation [1]. Greater attachment security in adulthood is longitudinally associated with greater life satisfaction, quality of life, physical health, and mental health [2,3]. Attachment security is also shown to protect against cognitive decline in later life [4]. The relevance of attachment security in human health and wellbeing across the lifespan is increasingly evident. The role of secure attachment in fostering human health and wellbeing across the lifespan underscores the importance of exploring its developmental origins and ongoing influences.

Parental sensitivity in infancy and childhood has been long considered an important aspect of attachment development, albeit not as exclusively responsible as was once thought [5]. Research also shows that the maternal–offspring attachment bond continues to play an important role during adolescence and not just early childhood [6,7,8], aligning with the evidence that finds that attachment remains vulnerable to change; that is, attachment security may strengthen or weaken beyond early childhood [9,10,11].

Beyond primary caregiving relationships, there are other interpersonal factors that can affect the development of attachment security. An ecological model of attachment considers broader relational influences such as wider family and social relationships [12]. Family conflict, and social relationships and supports across childhood and adolescence, for example, are shown to affect influence trajectories [12,13]. Positive interpersonal experiences with family members and friends/peers contribute to the construction of adaptive internal working models that guide interpersonal relationships [14,15,16]. Although attachment research has expanded its exploration of these broader interpersonal influences, the influence of the caregiver’s own social support network on their offspring’s attachment development is less studied. Maternal social support buffers against the impact of stressors on maternal wellbeing, thereby promoting sensitive and responsive caregiving behaviours [17]. Maternal social support is shown to improve maternal stress and maternal-infant attachment [18], and may be an important factor to consider when identifying the ways in which attachment security might be enhanced in offspring.

There have been increasing calls for a greater research focus on secure attachment and identifying the important factors in its development and ways in which it can be enhanced [11]. Few studies have prospectively examined the developmental antecedents of adult attachment, leaving a gap in understanding regarding the factors that shape attachment trajectories during periods of greater malleability prior to adulthood [5]. Understanding how family and peer relationships, social support networks, and the maternal–offspring relationship contribute to the development and maintenance of attachment security by early adulthood is an important area for future research.

The relational foundations of attachment, including maternal–child relationships and their broader relational networks, guided the focus of the current study. Utilising data from a prospective birth cohort, this study aimed to examine both maternal and offspring reported variables relating to the maternal–child relationship and broader child and family social relationships. Relevant data were collected at multiple timepoints throughout childhood and adolescence: 3–5 days post-partum (henceforward referred to as ‘birth’), childhood (5 years), adolescence (14 years), and early adulthood (21 years) and were examined in their association with offspring attachment security in early adulthood.

## 2. Materials and Methods

### 2.1. Sample

The study used data from a prospective birth cohort of mothers and their offspring who received antenatal care at an urban Australian hospital between 1981 and 1984. Of the 8556 private and public patient pregnant women invited to participate in the study, 8458 (98.9%) consented. Participants enrolled into the study provided informed written consent (see Najman et al. [19,20] for further details). Baseline data were collected on a total of 7223 singleton, live-birth offspring and their mothers. Mothers and offspring were followed prospectively when offspring were 6 months, 5, 14, and 21 years of age. The 21-year follow-up occurred from 2001 to 2004. Ethical approval for this study was obtained from The University of Queensland Human Research Ethics Committee (mothers: B/555/SS/01/NHMRC-30 November 2001, offspring: B/660/SS/01/NHMRC-20 December 2001) and The Mater Human Research Ethics Committee (mothers: 505A–29 June 2002, offspring: 506A-15 July 2002).

### 2.2. Measures

#### 2.2.1. Attachment Style Questionnaire (ASQ)

At the 21-year follow-up, offspring attachment styles were assessed using the Attachment Style Questionnaire (ASQ) [21]. This study used the ‘confidence (in self and others)’ subscale from a short form of the ASQ [22], which includes seven items that assess the extent to which individuals are confident about themselves and about their relationships with others. Higher scores reflect greater confidence. The ASQ confidence subscale showed good internal reliability (Cronbach’s alpha [α] 0.80).

#### 2.2.2. Maternal Social Interaction and Isolation

Social interaction and isolation were assessed using the Interview Schedule for Social Interaction (ISSI) [23] at birth, and at the 5-year follow-up. Mothers responded to four items (e.g., How many close friends do you have?). Higher scores reflected greater social interaction (and therefore less social isolation). Item responses were summed and divided by the total number of items to produce a mean subscale score. The ISSI showed acceptable internal reliability (α 0.72).

#### 2.2.3. Maternal Acceptance and Involvement

Maternal acceptance and involvement were assessed at 14 years using the Parenting acceptance/involvement scale [24]. The measure comprised 6 items rated on a 3-point scale (1 = ‘usually true’ to 3 = ‘not true’) (e.g., “She helps me with my hobbies/interests when I ask”). A total of 67 participants responded to items with ‘no female parent’ and were subsequently marked as ‘not applicable’ on the maternal acceptance and involvement scale for the current study. Item responses were summed and divided by the total number of items to produce an average subscale score. The maternal acceptance and involvement showed questionable internal reliability for mothers (α 0.55) and acceptable internal reliability for offspring (α 0.70).

#### 2.2.4. Parental Care and Overprotection

The Parental Bonding Instrument (PBI) [25] is a 25-item self-report instrument designed to measure recalled experiences of being parented to the age of 16 years, administered to offspring at 21-year follow-up. The parental care subscale includes items such as “Spoke to me with a warm and friendly voice”, while the parental overprotection subscale includes items such as “Invaded my privacy” with responses ranging from 1 = ‘usually true’ to 3 = ‘not true’. Some items were reverse coded and scored so that higher scores reflected greater parental care and greater overprotection. The PBI showed good internal reliability (parental care subscale: α 0.90; overprotection subscale: α 0.85).

#### 2.2.5. Single Item Independent Variables

Maternal relational closeness and change in support: At birth, mothers were asked “In general, do you feel you have a close relationship with your relatives?”. Responses were rated on a 4-point scale from 1 = ‘very close’ to 4 = ‘no relationship at all’. At five-years, mothers were asked “Has the number of people you can turn to for help/support changed in the last 5 years?” with the response options being 1 = ‘less people’, 2 = ‘same number’, and 3 = ‘more people’.

Close child friendships social interaction: At the 5 and 14-year follow-ups, mothers were asked “How many close friends does your child have?”. Responses were rated on a 4-point scale from 1 = ‘none’ to 4 = ‘4 or more’. In follow-up to the previous item, mothers were asked at the 5-year follow-up only “How many times per week does your child play with them?” with the response options being 1 = ‘once a week’, 2 = ‘1–2 times a week’, or 3 = ‘3+ times a week’.

Maternal–child physical touch and quality time: At the 14-year follow-up, mothers and offspring were independently asked “How often do you/your female parent spend time just talking with you/your child?” and “How often do you/your female parent hug you/your child?”. Responses were rated on a 4-point scale from 1 = ‘almost every day’ to 4 = ‘almost never’ and reverse coded to reflect the direction of the other study variables.

Child family satisfaction: At the 14-year follow-up, offspring were asked “How satisfied are you with your family life, that is the time you spend and the things you do with members of your family?”. Responses were rated on a 4-point scale from 1 = ‘very satisfied’ to 4 = ‘very dissatisfied’ and reverse coded to reflect the direction of the other study variables.

The full set of items used for the current study can be found in Supplementary Table S1.

### 2.3. Study Sample

A total of 3648 participants (female: *n* = 1931 52.9%) had no missing data on the outcome measure (ASQ confidence subscale) for the current study. The mean age of participants was 20.6 years (SD 0.9, range 18.2–24.3).

### 2.4. Statistical Analysis

All analyses were conducted in R (Version 4.1.1) [26]. The Lavaan package version 0.6-16 [27] was used to conduct a path analysis to assess pathways between study variables and confidence across timepoints from birth to 21 years. All variables were treated as continuous for the current study, which is a requirement of path analysis. To first determine the path model, Spearman’s nonparametric correlation coefficients were calculated among all study variables to evaluate their associations. The Comparative Fit Index (CFI ≥ 0.95), Tucker–Lewis Index (TLI ≥ 0.95) root mean square error of approximation (RMSEA ≤ 0.06), and standardised root mean square residual (SRMR ≤ 0.08) were used to assess model fit [28]. Missing data were assessed based on the full set of independent variables of interest. This process was repeated if one or more variables were not selected for inclusion in the path analysis. A sensitivity analysis was also conducted using a subset of participants with no missing data to test the robustness of the path model.

## 3. Results

### 3.1. Study Variable Correlations

Spearman’s rank correlations and descriptive statistics (mean, standard deviation, and range) for all independent variables are presented in Table 1. A path model was deemed appropriate based on theoretical associations and the strength of correlations between the independent variables shown in Table 1. Participant age and sex were considered covariates in the path model. However, they were not significantly associated with confidence (attachment security) and were therefore also not included in the model. Three 14-year follow-up variables had both maternal and offspring reports: maternal acceptance and involvement, maternal–child physical touch, and quality time. Offspring-reported variables were retained for the path analysis, as they were more strongly associated with the ASQ confidence scale at 21 years than maternally reported variables, which were subsequently not included.

### 3.2. Missing Data

The methods for addressing missing data are reported in Supplementary Figures S1 and S2. In brief, approximately half of the total sample (*n* = 1889, 51.8%) had missing data on one or more of the 16 independent variables of interest. The majority of missing data were attributable to around a third (*n* = 1140, 32.8%) of participants who did not participate in the 14-year follow-up. Path analysis was performed using full information maximum likelihood estimation (FIML). This advanced missing data method takes into account all observed data, and while missing values are not replaced or imputed, they are handled within the analytic model [29]. FIML reduces bias and improves efficiency compared to listwise deletion by using observed data to inform estimates for cases with missing values, under the assumption that data are missing at random. This method helps preserve statistical power and minimises potential distortions in parameter estimates that could arise from excluding incomplete cases.

### 3.3. Path Analysis

**Figure 1:**
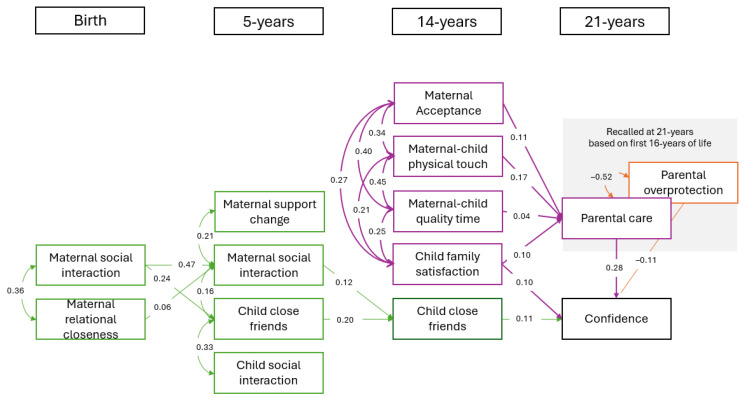
Path analysis with complete sample showing significant standardised regression coefficients (*p* < 0.05). Note. Green indicates child and family social relations pathway (all maternal reports). Purple indicates attuned caregiving pathway (all offspring reports). Orange indicates parental overprotection pathway.

Overall, this model fit the data well (X2(91) = 6084.10, *p* < 0.001, CFI = 0.96, TLI = 0.95, RMSEA = 0.03, and SRMR = 0.04). A path analysis was used to assess the effects of various interpersonal maternal and offspring-reported variables from birth through to 21 years on confidence, a measure of attachment security, at 21 years. Standardised regression coefficients for the path model are presented in Figure 1. The analysis identified two main explanatory pathways.

The first broad pathway was distinguished by maternally reported child and family social relation variables from birth through to adolescence. Maternal social interaction at birth was associated with maternal social interaction and child’s close friends at 5 years (β = 0.47, *p* < 0.001; β = 0.24, *p* < 0.001, respectively), which in turn, was associated with the maternally reported number of child’s close friends at 14 years (β = 0.12, *p* < 0.001; β = 0.20, *p* < 0.001, respectively), which was associated with confidence at 21 years (β = 0.11, *p* < 0.001).

The second broad pathway identified was distinguished by child-reported attuned caregiving variables in adolescence. Mother–child physical touch, mother–child quality time, maternal acceptance and involvement, and child family satisfaction were associated with recalled experiences of parental care up to 16 years (measured at 21 years), which, in turn, was associated with confidence in young adulthood (β = 0.28, *p* < 0.001). In addition, adolescent family satisfaction was also associated with confidence at 21 years (β = 0.10, *p* < 0.001).

Recalled parental overprotection up to 16 years (measured at age 21) was negatively correlated with confidence at age 21 (β = −0.11, *p* < 0.001) and with parental care (β = −0.52, *p* < 0.001).

The model explained 15.8%, 9.2%, 6.8%, 24.6%, and 5.6% of variance in the confidence, recalled parental care, number of child’s friends at age 14, maternal social interaction at age 5, and number of child’s friends at age 5, respectively.

### 3.4. Sensitivity Analysis

A follow-up sensitivity analysis was conducted to assess the effect of missing data on the path model. The path analysis was re-run with subset of 1911 participants with no missing data. The model fit was similar to that of the full sample (X2(91) = 6084.10, *p* < 0.001, CFI = 0.96, TLI = 0.95, RMSEA = 0.03, SRMR = 0.04). The standardised regression coefficients for the final path model are presented in Supplementary Figure S3. Results were similar to the full sample model.

## 4. Discussion

This study is one of only a few that examine the interpersonal antecedents of attachment security, as measured by the confidence in self and others subscale of the ASQ, longitudinally across childhood and adolescence. Two broad pathways were identified. First, greater maternally reported child and family social relations at birth, in early childhood, and during adolescence predicted greater offspring attachment security in early adulthood. Second, offspring who reported attuned parenting in adolescence (family satisfaction, quality time, physical touch, and maternal acceptance and involvement) and parental care retrospectively in early adulthood (but not parental overprotection) predicted greater attachment security in early adulthood. Lastly, recalled parental overprotection was directly negatively associated with offspring attachment security in early adulthood. These pathways are discussed in the following sections.

### 4.1. Child and Family Social Relations

The current study’s findings underscore the critical role of early maternal family relationships and social interactions in shaping attachment security during early adulthood. Previous research shows that parents who effectively mobilise social support for themselves provide more support to their children, promoting better mental health outcomes [30]. It is also important to recognise the bidirectional relationship between attachment and social relationships, where secure attachment may facilitate the development of adaptive social skills, leading to positive social interactions that reinforce and generalise attachment security to other relationships beyond the maternal–child relationship [31]. Although statistically significant, the effect sizes observed were relatively small, with only 6.8% of the variance in the number of the child’s close friends at age 14 explained by earlier mother and child familial and social relationships. This suggests that additional nuanced factors beyond the mere quantity of friendships, such as friendship quality and experiences, may further elucidate the influence of social connections on attachment [32].

Changes in maternal social support during the first five years were measured in the current study and were not related to offspring attachment at age 21; however, they were directly correlated with the level of maternal social interaction at age 5, which was associated with offspring attachment via the number of friends the child had at age 14. While peer relationships are highly imperative to healthy development, they are also only one aspect of a myriad of factors that influence attachment. Nonetheless, the current study’s findings align with what is already known regarding the importance of social connection in mother and child wellbeing. Enhancing the social support of mothers when they are raising their young children is known to improve the quality of their romantic relationships as well as the relationships with their offspring, all of which have positive implications for healthy child development [33].

### 4.2. Attuned Caregiving

Attuned caregiving experiences during adolescence predicted attachment security in early adulthood. Attuned parenting during adolescence is suggested to have a unique influence on the development of confidence in self and in others, separate to childhood experiences of attuned parenting [34]. Aspects of the maternal–adolescent bond, including frequency of physical touch (‘hugging’), quality time together (‘just talking’), and maternal acceptance and involvement, emerged as important predictors of offspring-reported attachment security via recollections of parental (or primary caregiver) care in early adulthood.

The level of child-reported family satisfaction at age 14 also predicted confidence via recollections of parental care; however, a direct effect on confidence was also observed. The finding of a direct effect provides evidence for the importance of overall family functioning and family relationships in the development of attachment security in addition to the mother–adolescent bond. Prior studies have shown that lower quality mother–offspring relationships are associated with early departure from the family home [35], while a higher quality relationship is associated with greater life satisfaction at age 30 [36]. Previous research on the study sample shows that early departure from the parental home and attachment security in early adulthood are significant predictors of life satisfaction at age 30 [2]. This offers a plausible explanation for the mechanisms by which a higher quality mother–adolescent relationship leads to increased life satisfaction longitudinally in adulthood.

Parental overprotection was the only variable to negatively predict the level of attachment security in early adulthood and, although moderately correlated with recollections of parental care, it was not strongly predicted by any of the other study variables. Previous research on young adults found that parental attachment anxiety is related to the parents’ overprotection through the level of separation anxiety they themselves experience [37]. The findings of the current study, however, indicate that the level of parental care has a greater influence on the offspring’s attachment security in early adulthood than overprotection. Taken together, high parental care and low overprotection, are shown to predict more secure attachment [38,39,40].

### 4.3. Implications

Numerous reviews link attachment with mental illness [41,42,43], child maltreatment [44], and domestic and family violence [45,46], all of which contribute to a substantial health and economic burden [47,48,49]. Prevention and early intervention efforts during early life can foster attachment security into adulthood and positively alter lifelong trajectories. Longitudinal research on mothers and offspring has demonstrated that maternal sensitivity leads to greater offspring social competence, which, in turn, predicts greater parental sensitivity in offspring when they themselves become parents [50]. Interventions targeting maternal sensitivity at the individual level have been developed [51]; however, findings from the current study indicate that broader public health and policy initiatives aimed at strengthening social support systems for caregivers and their children from early childhood may also lead to long-term improvements in the attachment security of the offspring. Such initiatives might incorporate societal systems such as early childcare, flexible working arrangements, and health and education systems [52,53,54], which play a pivotal role in supporting families and promoting social connection. Population-level strategies that increase parenting skills for adolescence as well as early childhood may also be an effective approach to increasing attachment security [55].

### 4.4. Limitations

Some caveats should be noted when interpreting the findings of the current study. First, the confidence (in self and others) subscale of the ASQ was used to assess attachment security. The subscale has been criticised as an incomplete measure of attachment security, as it does not capture comfort with intimacy, which is a central characteristic of attachment security [56]. Despite this, the confidence subscale is strongly negatively correlated with the ASQ’s discomfort with closeness, need for approval, and preoccupation with relationships subscales within the study sample [19]. However, the use of a more comprehensive measure of attachment security may yield different results. Second, the cross-sectional nature of recalled caregiving experiences and attachment outcomes limits causal inference for this part of the path analysis. Retrospective self-reports of caregiving experiences may also be influenced by current parent–child relationship quality. Regardless, the results underscore the salience of caregiving experiences in shaping attachment representations. Third, items within the MUSP study that captured elements of interpersonal relating were selected for inclusion in the current study; however, only two items—maternal social interaction and the maternally reported number of the child’s close friends—were repeated at multiple follow-up waves of the MUSP cohort. Consistent repeated measures across follow-ups may have provided greater insights into how each of the pathways identified (attuned caregiving and social relations) interact at different stages of childhood and adolescence. The longitudinal nature of the study also means that there is a potential mismatch between the historical context in which the data were collected and contemporary societal dynamics. Moreover, MUSP is a study of mothers and offspring; no paternal/second caregiver-reported information was captured. Lastly, approximately half of the study sample was lost to attrition at the 21-year follow-up. Previous research shows, however, that differential attrition rarely affected the estimates of association [57]. The results of the sensitivity analysis using only complete cases also showed no differences with the main analysis, which included participants who did not participate at the 14-year follow-up.

## 5. Conclusions

The current study identified that attachment security in early adulthood, measured through confidence in self and others, may be fostered through enhancing child and family social relations from birth to adolescence and through fostering parental attunement during childhood and adolescence. While the effect sizes observed were generally small in magnitude, the findings provide valuable insights into the interpersonal antecedents of adult attachment security and underscore the importance of promoting supportive social environments and sensitive caregiving practices to promote secure attachment, and ultimately healthy relational development into adulthood.

## Figures and Tables

**Table 1:** Spearman’s rank correlations between study variables.

		Birth		5 Years				14 Years								21 Years		
		1	2	3	4	5	6	7	8	9	10	11	12	13	14	15	16	17
**Birth**	1. Social interaction [M]																	
	2. Close with family [M]	**0.33**																
**5 years**	3. Social interaction [M]	**0.49**	**0.23**															
	4. Child close friends [M]	**0.25**	**0.09**	**0.29**														
	5. How often play [M]	**0.04**	0.02	**0.07**	**0.26**													
	6. Change in support [M]	**0.12**	**0.08**	**0.25**	**0.10**	0.03												
**14 years**	7. Accept/involve [M]	**0.06**	**0.07**	0.03	0.03	**0.04**	0.04											
	8. Child close friends [M]	**0.15**	**0.08**	**0.17**	**0.24**	**0.09**	**0.04**	0.02										
	9. How often talk [M]	**0.04**	**0.05**	0.01	**0.04**	**0.06**	−0.01	**0.20**	**0.10**									
	10. How often hug [M]	**0.06**	**0.07**	**0.04**	**0.07**	0.01	0.03	**0.12**	**0.07**	**0.31**								
	11. Accept/involve [O]	**0.08**	**0.10**	0.04	0.03	0.01	0.00	**0.23**	0.03	**0.13**	**0.15**							
	12. How often talk [O]	0.00	**0.06**	−0.01	0.00	0.01	0.00	**0.12**	**0.05**	**0.22**	**0.20**	**0.36**						
	13. How often hug [O]	0.03	**0.08**	0.00	−0.02	−0.02	0.00	**0.10**	0.03	**0.17**	**0.56**	**0.31**	**0.43**					
	14. Family satisfaction [O]	**0.05**	**0.06**	**0.06**	0.02	0.02	0.00	**0.08**	**0.07**	**0.14**	**0.13**	**0.26**	**0.25**	**0.22**				
**21 years**	15. Care [O]	**0.08**	**0.07**	**0.07**	−0.01	−0.01	**0.05**	**0.09**	**0.06**	**0.10**	**0.19**	**0.25**	**0.22**	**0.26**	**0.23**			
	16. Overprotection [O]	**−0.04**	0.00	**−0.04**	0.01	−0.01	**0.04**	**−0.04**	**−0.05**	−0.02	0.01	**−0.08**	**−0.10**	−0.03	**−0.12**	**−0.51**		
	17. Confidence [O]	**0.10**	**0.07**	**0.09**	**0.04**	0.04	0.03	**0.08**	**0.11**	**0.05**	**0.05**	**0.15**	**0.13**	**0.14**	**0.21**	**0.40**	**−0.28**	
	Mean	3.7	3.2	3.2	3.3	2.7	2.2	2.5	3.4	3.4	3.3	2.5	3.2	3.0	3.2	3.4	3.1	4.5
	SD	1.0	0.6	0.9	0.7	0.6	0.6	0.3	0.6	0.7	1.0	0.4	0.9	1.1	0.6	0.5	0.5	0.7
	Range	1.0–6.0	1.0–4.0	1.0–6.0	1.0–4.0	1.0–3.0	1.0–3.0	1.3–3.0	1.0–4.0	1.0–4.0	1.0–4.0	1.0–3.0	1.0–4.0	1.0–4.0	1.0–4.0	1.1–4.0	1.0–4.0	1.4–6.0

Note. Figures in bold = significant at the *p* < 0.05 level. SD = standard deviation. [M] = maternal report. [O] = offspring report. Variables in grey not included in the path analysis.

## Data Availability

The data that support the findings of this study are available upon request.

## Supplementary Materials

The following supporting information can be downloaded at: https://www.mdpi.com/article/10.3390/children12020255/s1. Figure S1: Missing data; Figure S2: Missing data for study variables; Figure S3: Path analysis with complete case subset (n = 1911) showing significant standardised regression coefficients (*p* < 0.05); Table S1: Study variables: measures and items.
