# Supplementary material for: The Interpersonal Antecedents of Attachment Security in Early Adulthood
Source: Children (Basel). 2025 Feb 19;12(2):255. doi: 10.3390/children12020255 (PMC11854251; doi:10.3390/children12020255)
Supplement: Supplementary file 1 [file children-12-00255-s001.zip › children-3477412-supplementary.pdf]

After selecting the variables to be included in the path analysis, missing data were re-examined based on the selected 13 independent variables. A total of 1,737 (47.6%) participants had missing data on at least one independent variable. To assess potential missing data patterns, a binary missing data variable was created (‘missing data’/ ‘complete data’). Logistic regressions were then run for each of the study variables selected for path analysis using the missing data variable as the dependent variable. Of the 13 independent variables, there was a small increased odds in missing data for participants with higher maternally reported closeness with relatives at birth (odds ratio [OR] = 1.11 95% CI 1.00 – 1.23), child reported higher family satisfaction (OR = 1.21, 95% CI 1.04 – 1.39), higher maternally reported child number of friends at 14-years (OR = 1.22, 95% CI 1.09 – 1.35) and higher child-reported recalled parental care at 21 (OR = 1.26, 95% CI 1.12 – 1.43). No differences between males and females were found. Results did not provide strong evidence for missing patterns. Missing data patterns are shown in Supplementary Figure 2.

Figure S1. Missing data.

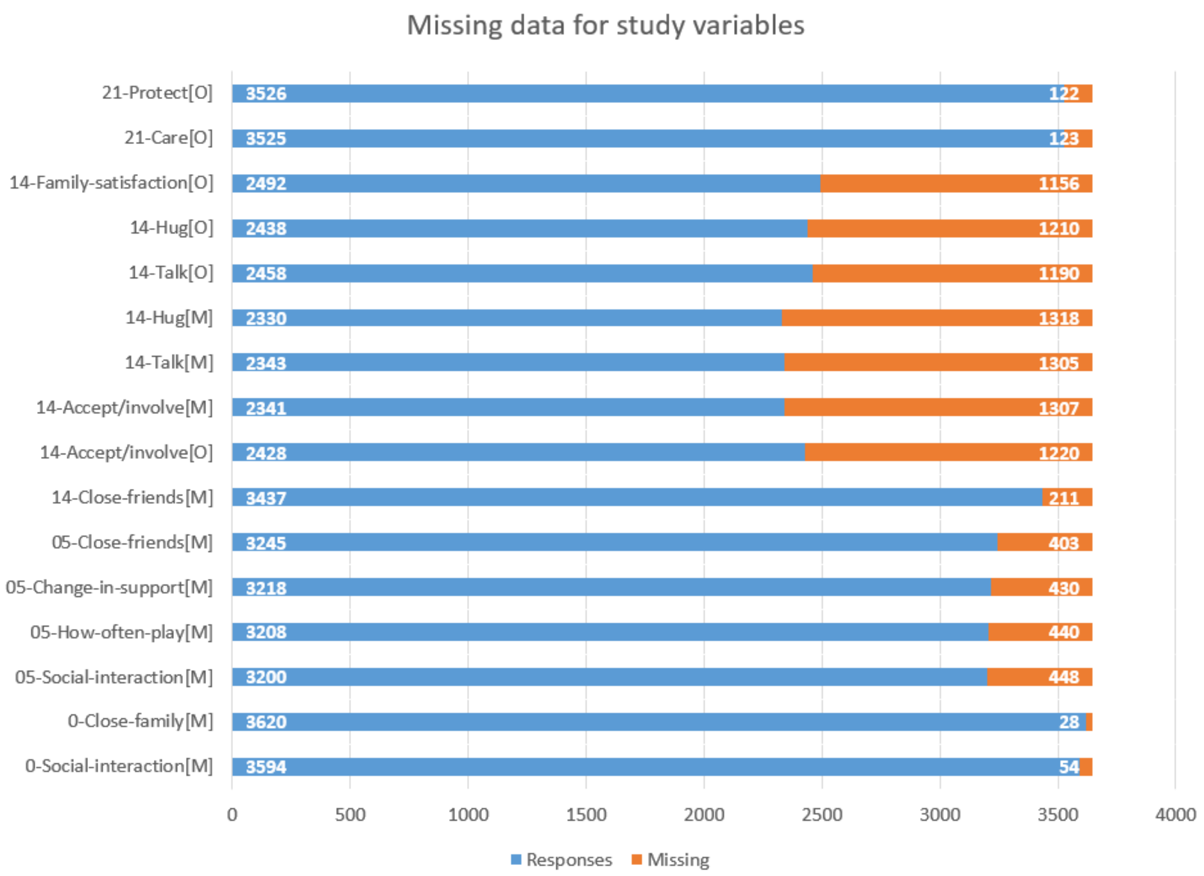

Figure S2. Missing data for study variables.

Note. [O] = offspring report. [M] = maternal report. Blue = responses. Orange = missing.

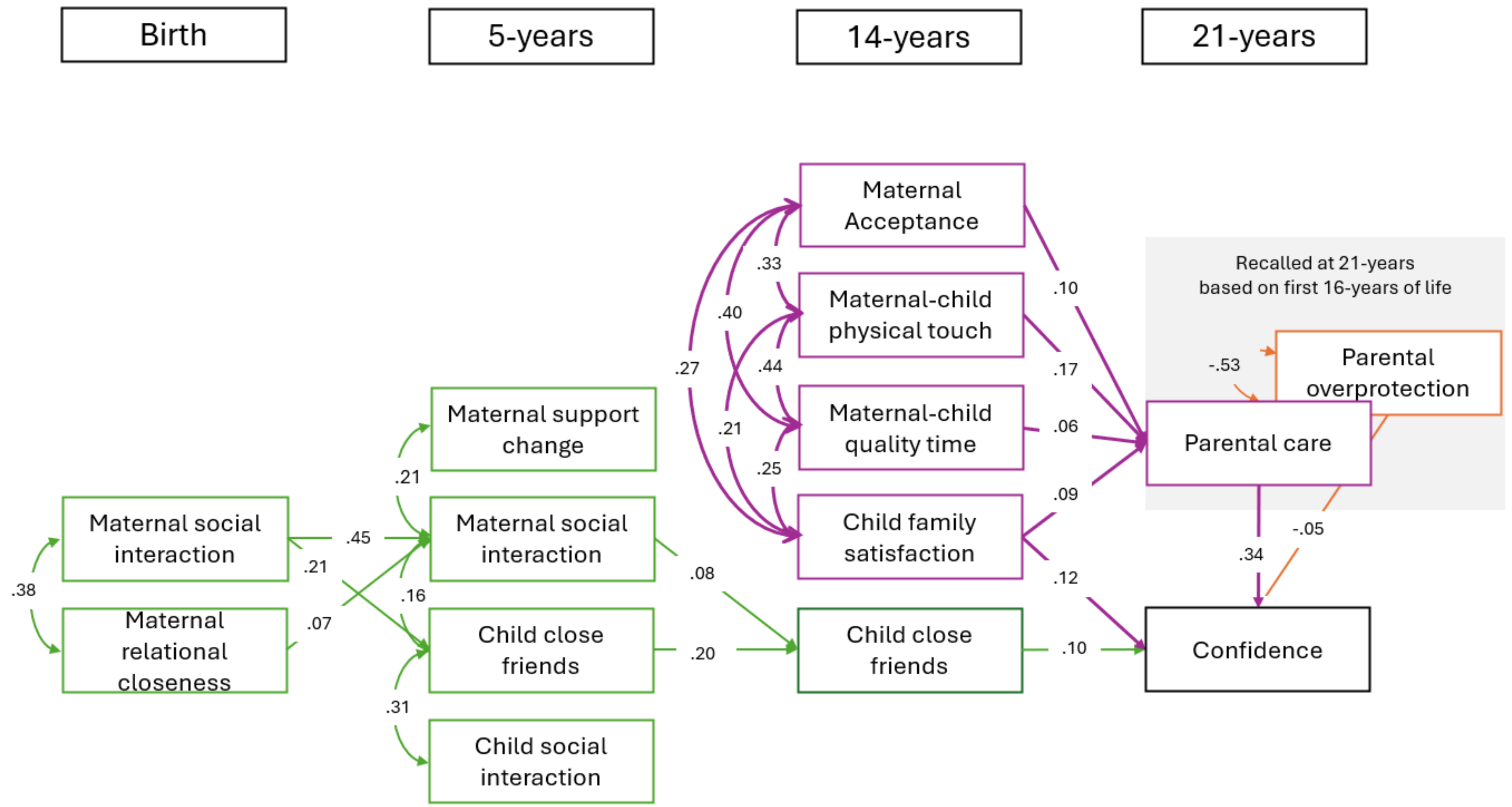

Figure S3. Path analysis with complete case subset (n=1,911) showing significant standardized regression coefficients (p<0.05).

Note. Green indicates child and family social relations pathway (all maternal report). Purple indicates attuned caregiving pathway (all offspring report). Orange indicates parental overprotection pathway. A direct path from maternal acceptance and involvement to confidence was tested but was not significant.

Table S1. Study variables: measures and items.

Child-report items
**Attachment Style Questionnaire confidence subscale: 21-years**
1. Overall, I am a worthwhile person.
2. I am easier to get to know than most people.
3. I feel confident that other people will be there for me when I need them.
4. I find it relatively easy to get close to other people.
5. I feel confident about relating to others.
6. I often worry that I do not really fit with other people*
7. I am confident that other people will like and respect me.
**Parental Bonding Instrument: 21-years**
1. Spoke to me with a warm and friendly voice [C]
2. Did not help me as much as I needed* [C]
3. Let me do those things I liked doing* [OP]
4. Seemed emotionally cold to me* [C]
5. Appeared to understand my problems [C]
6. Was affectionate to me [C]
7. Liked me to make my own decisions* [OP]
8. Did not want me to grow up [OP]
9. Tried to control everything I did [OP]
10. Invaded my privacy [OP]
11. Enjoyed talking things over with me [C]
12. Frequently smiled at me [C]
13. Tended to baby me [OP]
14. Did not seem to understand what I needed or wanted* [C]
15. Let me decide things for myself* [OP]
16. Made me feel I wasn't wanted* [C]
17. Could make me feel better when I was upset [C]
18. Did not talk with me very much* [C]
19. Tried to make me dependent on her/him [OP]
20. Felt I could not look after myself unless she/he was around [OP]
21. Gave me as much freedom as I wanted* [OP]
22. Let me go out as often as I wanted* [OP]
23. Was overprotective of me [OP]
24. Did not praise me* [C]
25. Let me dress any way I pleased* [OP]
**Maternal involvement and acceptance: 14-years**
1. I can count on her to help me out if I have some kind of problem.
2. She keeps pushing me to do my best in whatever I do.
3. She keeps pushing me to think for myself.
4. She helps me with my schoolwork.
5. When she wants me to do something, she explains why.
6. She helps me with my hobbies/interests when I ask.
**One-item measures: 14-years**
1. How often does your female parent spend time just talking with you? (labelled maternal-child quality time)
2. How often does your female parent hug you? (labelled maternal-child physical touch)

3. How satisfied are you with your family life, that is the time you spend and the things you do with members of your family? (labelled child family satisfaction)

Maternal-report items

Interview Schedule for Social Interaction: birth and 5-years

1. How many close friends do you have?
2. How many friends do you have whom you could visit at any time without waiting for an invitation? *(birth only)*
3. How many of your relatives do you see regularly?
4. To how many relatives do you feel really close?

Maternal involvement and acceptance: 14-years

1. My child can count on me to help me out if he/she has some kind of problem.
2. I keep pushing my child to do his/her best in whatever they do.
3. I keep pushing my child to think for themselves.
4. I help my child with his/her schoolwork.
5. When I want my child to do something, I explain why.
6. I help my child with his/her hobbies/interests when they ask

One-item measures

1. In general, do you feel you have a close relationship with your relatives? (birth) (labelled maternal relational closeness)
2. How many close friends does your child have? (5- and 14-years) (labelled child close friends)
3. How many times week does child play with them (5-years) (labelled child social interaction)
4. How often do you spend time just talking with your child (14-years) (labelled maternal-child quality time)
5. How often do you hug your child (14-years) (labelled maternal-child physical touch)
6. Has the number of people you can turn to for help/support changed in the last 5-years (5-years) (labelled maternal support change)

Note. *Item reverse scored [C] = Care subscale, [OP] = Overprotection subscale
